# 1-Chloro­methyl-4-nitro­benzene

**DOI:** 10.1107/S1600536810022191

**Published:** 2010-06-16

**Authors:** Aziz-ur-Rehman, Ammar Yasir, Mehmet Akkurt, Muhammad Athar Abbasi, Muhammad Jahangir, Islam Ullah Khan

**Affiliations:** a Department of Chemistry, Government College University, Lahore 54000, Pakistan; b Department of Physics, Faculty of Arts and Sciences, Erciyes University, 38039 Kayseri, Turkey

## Abstract

In the title compound, C_7_H_6_ClNO_2_, the nitro group is almost coplanar with the aromatic ring [dihedral angle = 2.9 (2)°], but the Cl atom deviates from the ring plane by 1.129 (1) Å. In the crystal, mol­ecules are linked by weak C—H⋯O inter­actions to generate chains.

## Related literature

For background on the toxicity of nitro-aromatic compounds, see: Moreno *et al.* (1986 ▶). For the synthesis of the title compound, see: Livermore & Sealock (1947 ▶). For bond-length data, see: Allen *et al.* (1987 ▶).
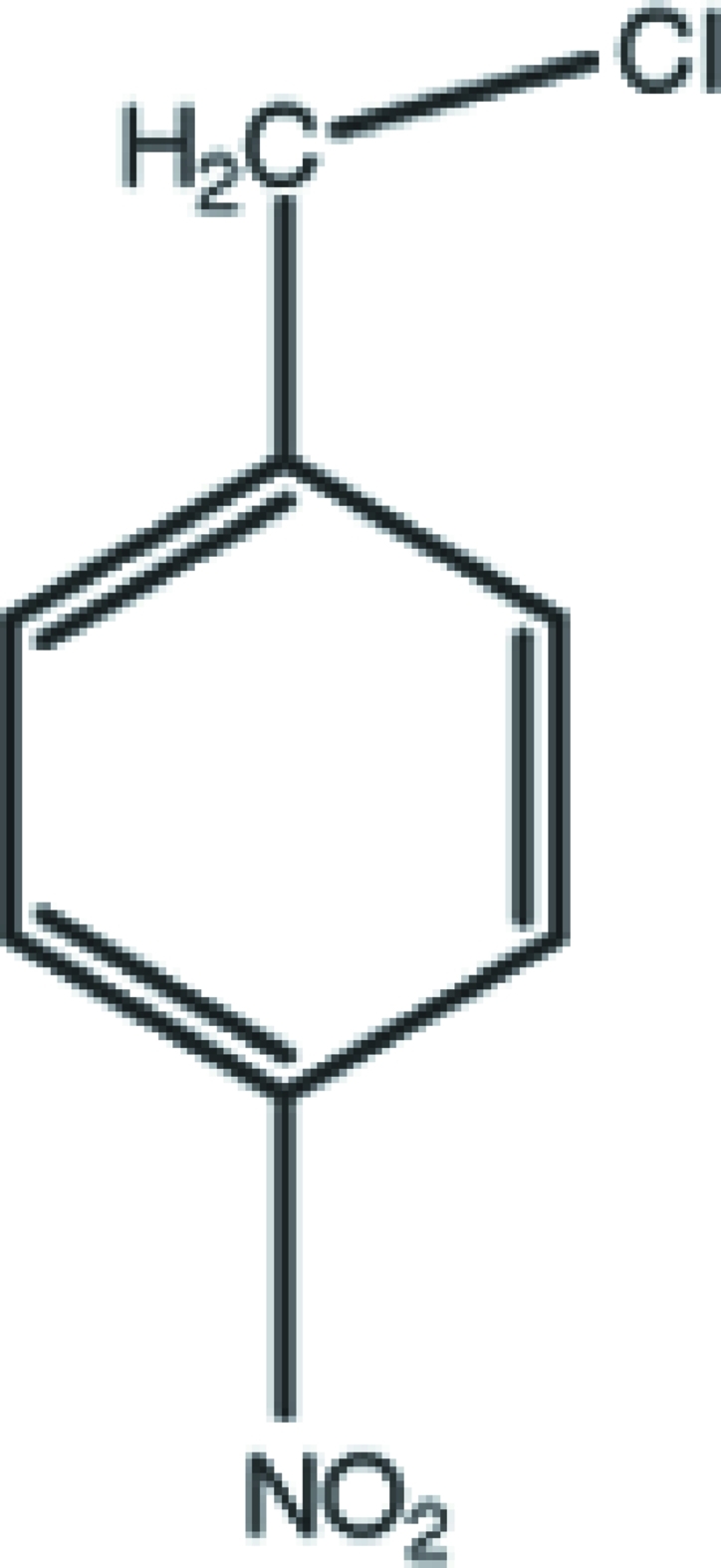


## Experimental

### 

#### Crystal data

C_7_H_6_ClNO_2_
*M* *_r_* = 171.58
Orthorhombic, 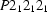

*a* = 4.7434 (1) Å
*b* = 6.4189 (2) Å
*c* = 24.9413 (11) Å
*V* = 759.40 (4) Å^3^
*Z* = 4
Mo *K*α radiation
μ = 0.45 mm^−1^
*T* = 296 K
0.35 × 0.11 × 0.10 mm

#### Data collection

Bruker APEXII CCD diffractometer
4389 measured reflections
1816 independent reflections
1586 reflections with *I* > 2σ(*I*)
*R*_int_ = 0.018

#### Refinement

*R*[*F* ^2^ > 2σ(*F* ^2^)] = 0.041
*wR*(*F* ^2^) = 0.103
*S* = 1.04
1816 reflections
100 parameters
H-atom parameters constrained
Δρ_max_ = 0.33 e Å^−3^
Δρ_min_ = −0.34 e Å^−3^
Absolute structure: Flack (1983 ▶), 662 Freidel pairs
Flack parameter: 0.02 (11)

### 

Data collection: *APEX2* (Bruker, 2007 ▶); cell refinement: *SAINT* (Bruker, 2007 ▶); data reduction: *SAINT*; program(s) used to solve structure: *SHELXS97* (Sheldrick, 2008 ▶); program(s) used to refine structure: *SHELXL97* (Sheldrick, 2008 ▶); molecular graphics: *ORTEP-3* (Farrugia, 1997 ▶); software used to prepare material for publication: *WinGX* (Farrugia, 1999 ▶), *PARST* (Nardelli, 1983 ▶) and *PLATON* (Spek, 2009 ▶).

## Supplementary Material

Crystal structure: contains datablocks global, I. DOI: 10.1107/S1600536810022191/hb5491sup1.cif

Structure factors: contains datablocks I. DOI: 10.1107/S1600536810022191/hb5491Isup2.hkl

Additional supplementary materials:  crystallographic information; 3D view; checkCIF report

## Figures and Tables

**Table 1:** Hydrogen-bond geometry (Å, °)

*D*—H⋯*A*	*D*—H	H⋯*A*	*D*⋯*A*	*D*—H⋯*A*
C7—H7*B*⋯O1^i^	0.97	2.48	3.396 (3)	158

Symmetry code: (i) 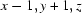.

**Fig. 1.**
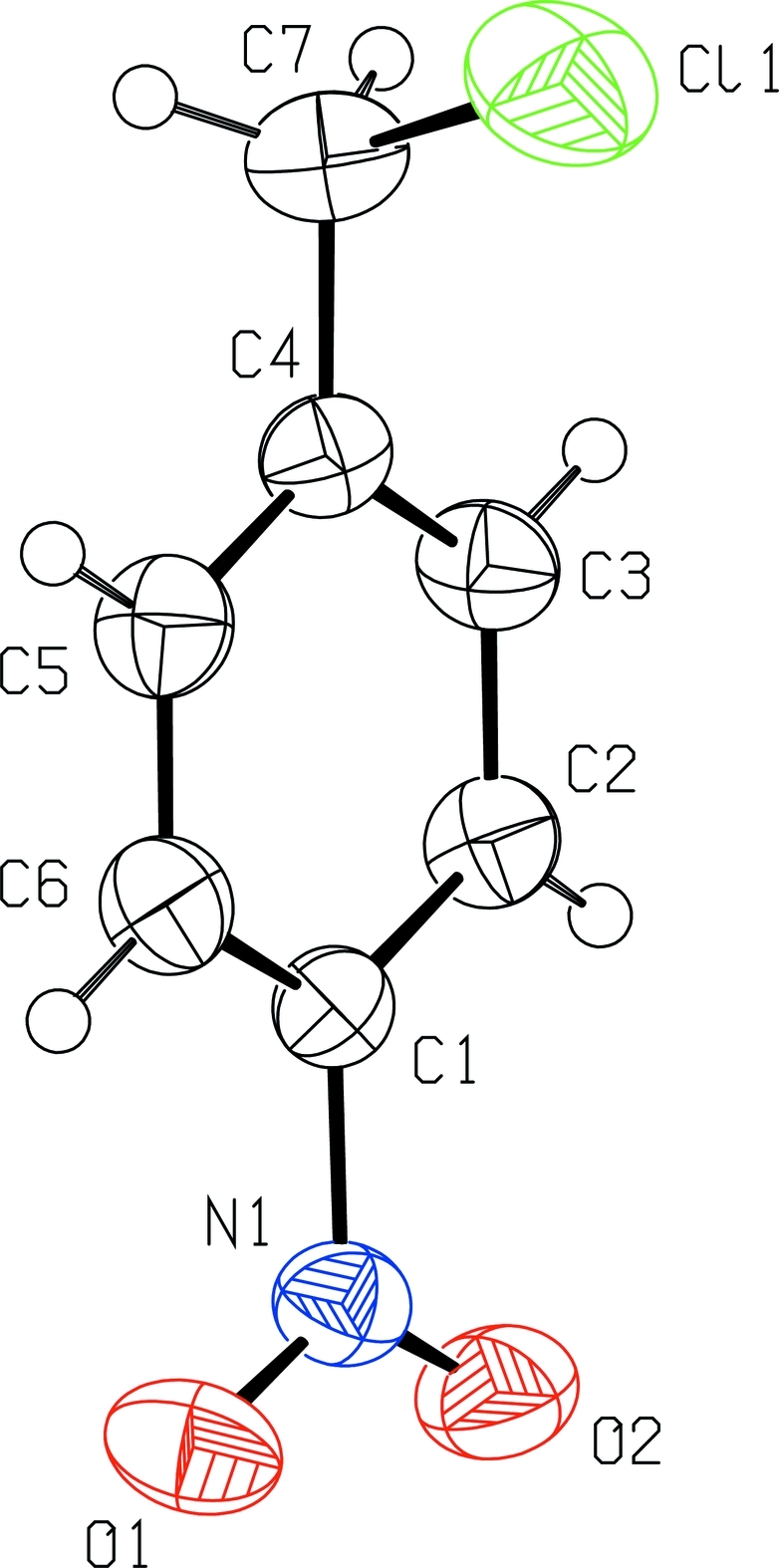
View of the title molecule, with displacement ellipsoids drawn at the 50% probability level.

**Fig. 2.**
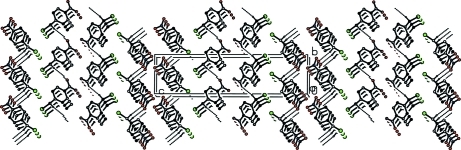
The crystal packing and the hydrogen bonding of (I) viewed down the a-axis. H-atoms not involved in hydrogen bonds have been omitted for clarity.

## supplementary crystallographic information

### Comment

The irreversible binding of the reductive intermediates of nitroaromatic compounds to protein and DNA is thought to be responsible for the carcinogenicity and mutagenicity of this class of compounds. Several studies revealed that some nitro radical metabolites with special features are expected to decompose to form neutral carbon-centered free radicals with not net reduction of the nitro group occurring. The radicals anions of *p*-and *o*-nitrobenzyl chloride are known to expel chloride to form the corresponding carbon-centered nitrobenzyl radicals with rate constants of 1 × 104 and 4 × 103 s^-1^. Such species are highly reactive and could account for the unusual cytotoxicity of these nitrocompounds (Moreno *et al.*, 1986). This structural report on 1-(chloromethyl)-4-nitrobenzene (*p*-nitrobenzyl chloride) might be helpful to carry out such studies on these nitroaromatic compounds in future.

The title molecule (I), (Fig. 1), is non-planar and the dihedral angle between the plane of the NO_2_ group and benzene (C1–C6) ring is 2.9 (2)°, while the C5—C4—C7—Cl1 torsion angle is 83.8 (2)°. In (I), the bond lengths (Allen *et al.*, 1987) and angles have values within the normal ranges.

In the crystal structure, there is no classic hydrogen bonds. A weak intermolecular C—H···O interaction contrubutes to the stability of the structure (Table 1, Fig. 2).

### Experimental

The title *p-*nitrobenzyl chloride was prepared by adding 5.3 ml of benzyl chloride slowly and with stirring to 27.5 ml of a mixture of equal parts of concentrated nitric and sulfuric acids cooled to 283 K. The temperature rose to 303 K during the 10 min required for the addition. The mixture was stirred for 30 min and then poured into 50 g of crushed ice. The crude material was recrystallized from ethanol. Product obtained was dissolved in ethanol and crystallized by slow evaporation of the solvent to yield colourless needles of (I) in an over-all yield of 46% (Livermore & Sealock, 1947).

### Refinement

H atoms were positioned geometrically (C—H = 0.93 and 0.97 Å) and allowed to ride on their parent atoms, with *U*_iso_(H) = 1.2*U*_eq_(C).

### Crystal data

**Table:**

C_7_H_6_ClNO_2_	*F*(000) = 352
*M**_r_* = 171.58	*D*_x_ = 1.501 Mg m^−^^3^
Orthorhombic, *P*2_1_2_1_2_1_	Mo *K*α radiation, λ = 0.71073 Å
Hall symbol: P 2ac 2ab	Cell parameters from 1957 reflections
*a* = 4.7434 (1) Å	θ = 3.3–26.7°
*b* = 6.4189 (2) Å	µ = 0.45 mm^−^^1^
*c* = 24.9413 (11) Å	*T* = 296 K
*V* = 759.40 (4) Å^3^	Needle, colourless
*Z* = 4	0.35 × 0.11 × 0.10 mm

### Data collection

**Table:**

Bruker APEXII CCD diffractometer	1586 reflections with *I* > 2σ(*I*)
Radiation source: sealed tube	*R*_int_ = 0.018
graphite	θ_max_ = 28.3°, θ_min_ = 3.3°
φ and ω scans	*h* = −5→6
4389 measured reflections	*k* = −8→5
1816 independent reflections	*l* = −33→17

### Refinement

**Table:**

Refinement on *F*^2^	Secondary atom site location: difference Fourier map
Least-squares matrix: full	Hydrogen site location: inferred from neighbouring sites
*R*[*F*^2^ > 2σ(*F*^2^)] = 0.041	H-atom parameters constrained
*wR*(*F*^2^) = 0.103	*w* = 1/[σ^2^(*F*_o_^2^) + (0.049*P*)^2^ + 0.1709*P*] where *P* = (*F*_o_^2^ + 2*F*_c_^2^)/3
*S* = 1.04	(Δ/σ)_max_ < 0.001
1816 reflections	Δρ_max_ = 0.33 e Å^−^^3^
100 parameters	Δρ_min_ = −0.34 e Å^−^^3^
0 restraints	Absolute structure: Flack (1983), 662 Freidel pairs
Primary atom site location: structure-invariant direct methods	Flack parameter: 0.02 (11)

### Special details

**Table:**

Geometry. Bond distances, angles *etc*. have been calculated using the rounded fractional coordinates. All su's are estimated from the variances of the (full) variance-covariance matrix. The cell e.s.d.'s are taken into account in the estimation of distances, angles and torsion angles
Refinement. Refinement on *F*^2^ for ALL reflections except those flagged by the user for potential systematic errors. Weighted *R*-factors *wR* and all goodnesses of fit *S* are based on *F*^2^, conventional *R*-factors *R* are based on *F*, with *F* set to zero for negative *F*^2^. The observed criterion of *F*^2^ > σ(*F*^2^) is used only for calculating -*R*-factor-obs *etc*. and is not relevant to the choice of reflections for refinement. *R*-factors based on *F*^2^ are statistically about twice as large as those based on *F*, and *R*-factors based on ALL data will be even larger.

### Fractional atomic coordinates and isotropic or equivalent isotropic displacement parameters (Å^2^)

**Table:**

	*x*	*y*	*z*	*U*_iso_*/*U*_eq_	
Cl1	0.75015 (18)	0.45250 (12)	0.22343 (3)	0.0812 (3)	
O1	1.4188 (4)	−0.3381 (3)	0.07002 (7)	0.0621 (6)	
O2	1.4761 (4)	−0.0854 (3)	0.01453 (7)	0.0601 (6)	
N1	1.3647 (3)	−0.1634 (3)	0.05351 (7)	0.0434 (5)	
C1	1.1532 (4)	−0.0398 (3)	0.08269 (7)	0.0364 (5)	
C2	1.0829 (4)	0.1540 (3)	0.06377 (7)	0.0421 (6)	
C3	0.8901 (4)	0.2704 (3)	0.09215 (8)	0.0437 (6)	
C4	0.7690 (4)	0.1928 (3)	0.13883 (7)	0.0393 (5)	
C5	0.8402 (5)	−0.0046 (3)	0.15629 (8)	0.0472 (6)	
C6	1.0341 (5)	−0.1235 (3)	0.12836 (8)	0.0459 (6)	
C7	0.5663 (5)	0.3213 (4)	0.17034 (8)	0.0533 (7)	
H2	1.16370	0.20560	0.03250	0.0510*	
H3	0.84040	0.40230	0.08000	0.0520*	
H5	0.75690	−0.05800	0.18710	0.0570*	
H6	1.08280	−0.25630	0.14000	0.0550*	
H7A	0.42040	0.23280	0.18530	0.0640*	
H7B	0.47710	0.42290	0.14710	0.0640*	

### Atomic displacement parameters (Å^2^)

**Table:**

	*U*^11^	*U*^22^	*U*^33^	*U*^12^	*U*^13^	*U*^23^
Cl1	0.0957 (5)	0.0831 (5)	0.0647 (4)	0.0041 (4)	0.0047 (4)	−0.0299 (3)
O1	0.0677 (11)	0.0514 (9)	0.0671 (10)	0.0222 (8)	−0.0024 (8)	0.0041 (8)
O2	0.0584 (10)	0.0629 (11)	0.0591 (9)	0.0072 (8)	0.0175 (8)	0.0010 (8)
N1	0.0412 (8)	0.0450 (9)	0.0440 (8)	0.0046 (8)	−0.0047 (7)	−0.0051 (7)
C1	0.0336 (8)	0.0382 (9)	0.0374 (8)	0.0008 (7)	−0.0048 (7)	−0.0001 (7)
C2	0.0446 (10)	0.0451 (11)	0.0366 (9)	0.0024 (9)	0.0012 (7)	0.0076 (8)
C3	0.0496 (11)	0.0409 (10)	0.0406 (9)	0.0079 (9)	−0.0016 (8)	0.0079 (8)
C4	0.0362 (9)	0.0460 (10)	0.0357 (8)	0.0024 (9)	−0.0033 (7)	−0.0008 (7)
C5	0.0526 (11)	0.0495 (12)	0.0394 (9)	−0.0012 (9)	0.0046 (8)	0.0080 (8)
C6	0.0500 (11)	0.0405 (10)	0.0471 (10)	0.0015 (9)	0.0001 (9)	0.0084 (8)
C7	0.0510 (12)	0.0613 (13)	0.0475 (11)	0.0111 (12)	0.0033 (9)	−0.0004 (10)

### Geometric parameters (Å, °)

**Table:**

Cl1—C7	1.795 (2)	C4—C7	1.491 (3)
O1—N1	1.222 (3)	C5—C6	1.383 (3)
O2—N1	1.215 (2)	C2—H2	0.9300
N1—C1	1.472 (3)	C3—H3	0.9300
C1—C2	1.372 (3)	C5—H5	0.9300
C1—C6	1.380 (3)	C6—H6	0.9300
C2—C3	1.377 (3)	C7—H7A	0.9700
C3—C4	1.391 (3)	C7—H7B	0.9700
C4—C5	1.382 (3)		
			
O1—N1—O2	123.83 (19)	C1—C2—H2	121.00
O1—N1—C1	118.12 (17)	C3—C2—H2	121.00
O2—N1—C1	118.05 (18)	C2—C3—H3	120.00
N1—C1—C2	118.98 (16)	C4—C3—H3	120.00
N1—C1—C6	118.51 (17)	C4—C5—H5	120.00
C2—C1—C6	122.51 (18)	C6—C5—H5	120.00
C1—C2—C3	118.48 (17)	C1—C6—H6	121.00
C2—C3—C4	120.69 (18)	C5—C6—H6	121.00
C3—C4—C5	119.43 (18)	Cl1—C7—H7A	110.00
C3—C4—C7	120.64 (18)	Cl1—C7—H7B	110.00
C5—C4—C7	119.93 (18)	C4—C7—H7A	110.00
C4—C5—C6	120.65 (18)	C4—C7—H7B	110.00
C1—C6—C5	118.22 (18)	H7A—C7—H7B	108.00
Cl1—C7—C4	109.58 (16)		
			
O1—N1—C1—C2	−177.90 (18)	C1—C2—C3—C4	−0.2 (3)
O1—N1—C1—C6	2.4 (3)	C2—C3—C4—C5	−1.0 (3)
O2—N1—C1—C2	2.4 (3)	C2—C3—C4—C7	178.30 (19)
O2—N1—C1—C6	−177.28 (19)	C3—C4—C5—C6	1.2 (3)
N1—C1—C2—C3	−178.37 (17)	C7—C4—C5—C6	−178.1 (2)
C6—C1—C2—C3	1.3 (3)	C3—C4—C7—Cl1	−95.5 (2)
N1—C1—C6—C5	178.55 (18)	C5—C4—C7—Cl1	83.8 (2)
C2—C1—C6—C5	−1.1 (3)	C4—C5—C6—C1	−0.2 (3)

### Hydrogen-bond geometry (Å, °)

**Table:**

*D*—H···*A*	*D*—H	H···*A*	*D*···*A*	*D*—H···*A*
C7—H7B···O1^i^	0.97	2.48	3.396 (3)	158

Symmetry codes: (i) *x*−1, *y*+1, *z*.

## References

[bb1] Allen, F. H., Kennard, O., Watson, D. G., Brammer, L., Orpen, A. G. & Taylor, R. (1987). *J. Chem. Soc. Perkin Trans. 2*, pp. S1–19.

[bb2] Bruker (2007). *APEX2* and *SAINT* Bruker AXS Inc., Madison, Wisconsin, USA.

[bb3] Farrugia, L. J. (1997). *J. Appl. Cryst.* **30**, 565.

[bb4] Farrugia, L. J. (1999). *J. Appl. Cryst.* **32**, 837–838.

[bb5] Flack, H. D. (1983). *Acta Cryst.* A**39**, 876–881.

[bb6] Livermore, A. H. & Sealock, R. R. (1947). *J. Biol. Chem.* **167**, 699–704. 20287900

[bb7] Moreno, S. N. J., Schreiber, J. & Mason, R. P. (1986). *J. Biol. Chem.* **261**, 7811–7815. 3011800

[bb8] Nardelli, M. (1983). *Comput. Chem.* **7**, 95–98.

[bb9] Sheldrick, G. M. (2008). *Acta Cryst.* A**64**, 112–122. 10.1107/S0108767307043930 18156677

[bb10] Spek, A. L. (2009). *Acta Cryst.* D**65**, 148–155. 10.1107/S090744490804362X PMC2631630 19171970

